# Effects of adjunctive brexpiprazole in patients with major depressive disorder and sleep disturbance: a *post hoc* analysis of three randomized trials

**DOI:** 10.3389/fpsyt.2025.1618176

**Published:** 2025-08-07

**Authors:** Ferhat Ardic, Zhen Zhang, Michael Hogan

**Affiliations:** ^1^ Medical Affairs, H. Lundbeck A/S, Copenhagen, Denmark; ^2^ Medical and Real-World Data Analytics, Otsuka Pharmaceutical Development & Commercialization Inc., Princeton, NJ, United States; ^3^ Medical Affairs, Otsuka Pharmaceutical Development & Commercialization Inc., Princeton, NJ, United States

**Keywords:** brexpiprazole, major depressive disorder, sleep disturbance, adjunctive, antidepressant, sleep disturbance factor

## Abstract

**Introduction:**

Sleep disturbances are common in major depressive disorder (MDD). This *post hoc* analysis aimed to evaluate the effects of adjunctive brexpiprazole in patients with MDD and sleep disturbance.

**Methods:**

Data were pooled from three placebo-controlled trials of adjunctive brexpiprazole in patients with MDD and inadequate response to antidepressant treatments (ADTs) (ClinicalTrials.gov identifiers: NCT01360645, NCT01360632, NCT02196506). Using the Hamilton Depression Rating Scale Sleep Disturbance Factor (SDF) (sum of three insomnia items), patients were categorized by high (SDF ≥4) or low (SDF <4) baseline sleep disturbance. Change in Montgomery–Åsberg Depression Rating Scale (MADRS) Total, SDF, and other efficacy scores were evaluated for ADT + brexpiprazole 2 or 3 mg versus ADT + placebo. Safety was assessed by the incidence of treatment-emergent adverse events (TEAEs).

**Results:**

At baseline, 689/1,160 (59.4%) patients had high sleep disturbance, and 471/1,160 (40.6%) had low sleep disturbance. At Week 6, ADT + brexpiprazole showed greater improvement in MADRS Total score versus ADT + placebo in both subgroups (high SDF: p<0.0001; low SDF: p=0.0058), and greater SDF score improvement in the high SDF subgroup (p=0.021). The incidence of TEAEs was higher with ADT + brexpiprazole than ADT + placebo in the high SDF subgroup (59.8%, 51.6%) and the low SDF subgroup (62.4%, 40.9%).

**Conclusion:**

Over 6 weeks, adjunctive brexpiprazole was associated with improved depression severity versus adjunctive placebo, regardless of baseline sleep disturbance. In patients with high baseline sleep disturbance, improvement in sleep disturbance was greater with adjunctive brexpiprazole versus adjunctive placebo, and was generally not accompanied by daytime sedation. No new safety signals were observed within each subgroup.

## 1 Introduction

Sleep disturbances such as insomnia (difficulty sleeping) and hypersomnia (excessive sleeping) are frequently reported by patients with major depressive disorder (MDD) (1–3), and are included in MDD diagnostic criteria (4). As a pervasive feature of depression, sleep disturbances often precede depressive episodes and can persist as residual symptoms during remission in patients taking antidepressant treatment (ADT) (1, 5–7). Insomnia is associated with increased severity and duration of depressive episodes, and increased risk of relapse (8). Insomnia is also a risk factor for developing treatment-resistant depression, and is 1.6 times more common in patients with inadequate response to treatment (9). Sleep disturbances in MDD negatively impact patients’ quality of life and executive functioning (3, 10). Improving sleep in patients with MDD is therefore crucial for improving overall patient outcomes (8, 11).

Brexpiprazole is an atypical antipsychotic that is approved in the United States, Canada, and various other countries (but not in the European Union) as adjunctive therapy for patients with MDD and inadequate response to ADTs (12, 13). Whereas diverse treatment strategies may be used in clinical practice (14), recent guidelines from the Canadian Network for Mood and Anxiety Treatments (CANMAT) recommend brexpiprazole (or aripiprazole) as the first-line adjunctive treatment in difficult-to-treat depression (15). Real-world studies provide evidence for the efficacy of adjunctive brexpiprazole in MDD, and potentially extending to other treatment resistance scenarios (16–21).

A previous exploratory open-label study suggested that adjunctive brexpiprazole may improve various sleep parameters in patients with MDD and sleep disturbances, including total sleep time, sleep efficiency, wake time after sleep onset, sleep onset latency, latency to persistent sleep, and circadian rhythm (22, 23). Furthermore, in randomized controlled trials, adjunctive brexpiprazole has shown efficacy versus adjunctive placebo on the sleep item of a depression rating scale (24). Thus, it may be hypothesized that adjunctive brexpiprazole can help patients with MDD and sleep-related symptoms. The aim of this *post hoc* analysis was to evaluate the effects of adjunctive brexpiprazole versus adjunctive placebo in patients with MDD (and inadequate response to ADTs) and sleep disturbance, using pooled data from three randomized controlled trials.

## 2 Methods

### 2.1 Study design and patients

This *post hoc* analysis included pooled data from three similarly designed, randomized, double-blind, placebo-controlled, Phase 3 trials of adjunctive brexpiprazole in adults with MDD and inadequate response to ADTs: Pyxis (Trial 228; ClinicalTrials.gov identifier: NCT01360645) (25), Polaris (Trial 227; NCT01360632) (26), and Sirius (Trial 214; NCT02196506) (27). All three trials were conducted in compliance with the International Council for Harmonisation Good Clinical Practice guideline and local regulatory requirements, and with the principles laid out in the Declaration of Helsinki. The protocols were approved by independent ethics committees, and all patients provided written informed consent to participate.

Detailed trial designs have been previously published (25–27). In brief, the studies enrolled outpatients aged 18–65 years with MDD as per the Diagnostic and Statistical Manual of Mental Disorders, Fourth Edition, Text Revision (DSM-IV-TR) criteria (28); a current depressive episode of ≥8 weeks; an inadequate response (defined as <50% improved) to 1–3 prior ADTs during the current episode; and a Hamilton Depression Rating Scale (HAM-D_17_) (29, 30) Total score of ≥18. Exclusion criteria included suicidal ideation or behavior, substance abuse or dependence, and specified DSM-IV-TR comorbidities. Comorbid DSM-IV-TR sleep disorders were not exclusionary.

In the trials, eligible patients received single-blind placebo together with an investigator-determined, open-label ADT (sertraline, escitalopram, duloxetine, fluoxetine, paroxetine controlled-release, or venlafaxine extended-release) for 8 weeks. The purpose of the 8-week prospective treatment phase was to identify patients with inadequate response to an additional ADT. Inadequate response to ADT was defined as <50% reduction in HAM-D_17_ Total score from the start to the end of the prospective treatment phase, HAM-D_17_ Total score of ≥14 at the end of the prospective treatment phase, <50% reduction in Montgomery–Åsberg Depression Rating Scale (MADRS) (31) Total score from the start of the prospective treatment phase to each scheduled visit, and a Clinical Global Impression – Improvement (CGI-I) (32) score of ≥3 at each scheduled visit during the prospective treatment phase.

Patients with an inadequate response to ADT were randomized to 6 weeks of double-blind treatment with fixed-dose brexpiprazole (1, 2, or 3 mg/day, depending on the study) or placebo, adjunctive to their continued ADT. Brexpiprazole was initiated at a dose of 0.5 mg/day, titrated to 1 mg/day after 1 week, and titrated to the allocated dose after 2 weeks. Concomitant benzodiazepines and non-benzodiazepine sleep aids were prohibited except for the short-term management of treatment-emergent agitation/anxiety and insomnia, respectively; these drugs could not be taken in the 12 hours before a scheduled efficacy or safety assessment.

### 2.2 Sleep disturbance subgroups

In this *post hoc* analysis, sleep disturbance was measured by the Sleep Disturbance Factor (SDF), which is the sum of three HAM-D_17_ item scores: item 4 “insomnia – early”, item 5 “insomnia – middle”, and item 6 “insomnia – late” (33, 34). Each item is rated on a 3-point scale: 0 (absent), 1 (occasional), and 2 (frequent). Scores for the three insomnia items are summed to form the SDF score, which ranges from 0 (no sleep disturbance) to 6 (maximum sleep disturbance) (29, 30, 34). Patients were categorized by baseline (the randomization visit, prior to dosing) level of sleep disturbance, defined as in previous literature as high (SDF score ≥4), or low (SDF score <4) (34). Patients with an SDF score of 0 (indicating no sleep disturbance) were also included in the low SDF subgroup.

### 2.3 Outcome measures

The primary efficacy endpoint in each of the three trials was change in MADRS (31) Total score from baseline to Week 6 of the randomized treatment phase. The MADRS, a measure of depression severity, was administered at baseline and at weekly intervals throughout the randomized phase, and was the main depression outcome of this *post hoc* analysis.

Change in depression severity was also assessed using the clinician-reported HAM-D_17_ (29, 30) Total score and Clinical Global Impression – Severity (CGI-S) (32) score. MADRS response rates (defined as a ≥50% reduction in MADRS Total score from baseline to Week 6) were also assessed. Change in functioning was assessed using the patient-reported Sheehan Disability Scale (SDS) (35, 36) score. In this *post hoc* analysis, change in sleep disturbance was assessed using the SDF score, and the MADRS “reduced sleep” item score. Rating scales were administered by trained and experienced clinicians, who were certified for the trials to administer the MADRS and HAM-D_17_. The number of raters within each trial center was kept to a minimum.

In this *post hoc* analysis, safety was assessed by the incidence of treatment-emergent adverse events (TEAEs), with a focus on sedating TEAEs (somnolence, fatigue, sedation, lethargy) and activating TEAEs (akathisia, restlessness, insomnia, initial insomnia, middle insomnia, terminal insomnia).

### 2.4 Data analysis

In this *post hoc* analysis, data were pooled for adjunctive brexpiprazole doses of 2 or 3 mg/day, reflecting the recommended-to-maximum brexpiprazole doses for the adjunctive treatment of MDD in the United States (12). Separately, data were pooled for adjunctive placebo.

Efficacy analyses were conducted for all patients randomized per final protocols who received at least one dose of double-blind medication and had both a baseline and at least one post-baseline MADRS Total evaluation in the randomized treatment phase. Safety analyses were conducted for all patients who received at least one dose of double-blind medication in the randomized treatment phase. Patients without a baseline HAM-D_17_ measurement could not be categorized into SDF subgroups, and were therefore excluded. Change in MADRS Total score, change in HAM-D_17_ Total score, change in CGI-S score, MADRS response rates, and change in SDS score were assessed in the high SDF and low SDF subgroups. Change in sleep endpoints – SDF score, and MADRS “reduced sleep” item score – were assessed in the high SDF subgroup, only.

Patient baseline demographic and clinical characteristics were summarized using descriptive statistics. For MADRS Total score, CGI-S score, SDS score, and MADRS “reduced sleep” item score, least squares (LS) mean changes from baseline were calculated using a mixed model for repeated measures (MMRM) method with model terms of study (to account for potential heterogeneity across studies), treatment, visit, treatment-by-visit and baseline-by-visit interaction. An unstructured covariance was used by default; normality and other covariance structures were examined by fitting the MMRM with alternative assumptions (such as t-distributed residuals/random effect, homogeneity or heterogeneity of variance, or autocorrelation over visits). For HAM-D_17_ Total score and SDF score, LS mean changes from baseline were calculated using an analysis of covariance (ANCOVA) model on the last observation carried forward (LOCF) dataset, with treatment and study center as the main effects and baseline value as the covariate. For MADRS response rates, the Cochran–Mantel–Haenszel association test, controlling for study site, was conducted using LOCF. All p-values were tested at a nominal 0.05 level (two-sided) with no adjustment for multiplicity. Cohen’s *d* effect sizes (37) were also calculated. The incidence of TEAEs were summarized using descriptive statistics.

Analyses were performed using SAS version 9.4 (SAS Institute Inc; Cary, NC).

## 3 Results

### 3.1 Patients

Data were analyzed for a total of 1,160 patients (efficacy and safety samples), of whom 689 (59.4%) were in the high SDF subgroup (ADT + brexpiprazole 2 or 3 mg/day, n=348; ADT + placebo, n=341) and 471 (40.6%) were in the low SDF subgroup (ADT + brexpiprazole 2 or 3 mg/day, n=229; ADT + placebo, n=242).

Baseline demographics and clinical characteristics were similar across the three trials (25–27). Within each pooled subgroup, baseline demographic and clinical characteristics were generally similar between ADT + brexpiprazole and ADT + placebo treatment groups (**Table 1**). Baseline depression severity was higher in the high SDF subgroup than in the low SDF subgroup, but was similar between treatment arms within each subgroup.

**Table 1:** Baseline demographic and clinical characteristics and concomitant medications in high SDF (≥4) and low SDF (<4) subgroups.

Characteristic	High SDF subgroup		Low SDF subgroup	
	ADT + placebo (n=341)	ADT + brexpiprazole (n=348)	ADT + placebo (n=242)	ADT + brexpiprazole (n=229)
Demographic characteristics				
Age (years), mean (SD)	45.0 (12.1)	44.1 (11.4)	44.3 (11.5)	43.5 (12.4)
Sex, n (%)				
Female	246 (72.1)	247 (71.0)	156 (64.5)	163 (71.2)
Male	95 (27.9)	101 (29.0)	86 (35.5)	66 (28.8)
BMI (kg/m^2^), mean (SD)	30.1 (7.2)	30.1 (6.9)	28.8 (6.8)	29.1 (6.7)
Race, n (%)				
Asian	2 (0.6)	1 (0.3)	4 (1.7)	0 (0)
Black or African American	49 (14.4)	48 (13.8)	20 (8.3)	11 (4.8)
White	285 (83.6)	287 (82.5)	214 (88.4)	213 (93.0)
Other	5 (1.5)	12 (3.4)	4 (1.7)	5 (2.2)
Clinical characteristics				
Duration of current episode (months), mean (SD)	16.8 (34.1)	14.5 (17.8)	17.4 (39.0)	16.1 (30.8)
Recurrent episode, n (%)	289 (84.8)	293 (84.2)	208 (86.0)	202 (88.2)
MADRS Total score, mean (SD)	27.8 (5.9)	27.9 (5.5)	24.9 (5.0)	25.1 (5.1)
HAM-D_17_ Total score, mean (SD)	22.4 (3.7)	22.6 (3.7)	19.3 (3.1)	19.6 (3.1)
CGI-S score, mean (SD)	4.3 (0.6)	4.3 (0.6)	4.1 (0.6)	4.1 (0.6)
SDS score, mean (SD)	6.0 (2.1) [n=336]	5.9 (2.3) [n=347]	5.6 (2.0) [n=240]	5.7 (2.1) [n=227]
SDF score, mean (SD)	4.8 (0.8)	4.9 (0.8)	2.1 (1.0)	2.2 (0.9)
Concomitant medications				
Took a medication with a sedative or hypnotic effect during the trial, n (%)^a^	75 (22.0)	90 (25.9)	39 (16.1)	43 (18.8)
Took a benzodiazepine during the trial, n (%)	27 (7.9)	37 (10.6)	16 (6.6)	13 (5.7)

a Including benzodiazepines, non-benzodiazepine sleep aids (e.g., zolpidem, zopiclone, eszopiclone), and antihistamines.

ADT, antidepressant treatment; BMI, body mass index; CGI-S, Clinical Global Impression – Severity; HAM-D_17_, 17-item Hamilton Depression Rating Scale; MADRS, Montgomery–Åsberg Depression Rating Scale; SD, standard deviation; SDF, Sleep Disturbance Factor; SDS, Sheehan Disability Scale.

### 3.2 Efficacy

**Figure 1:**
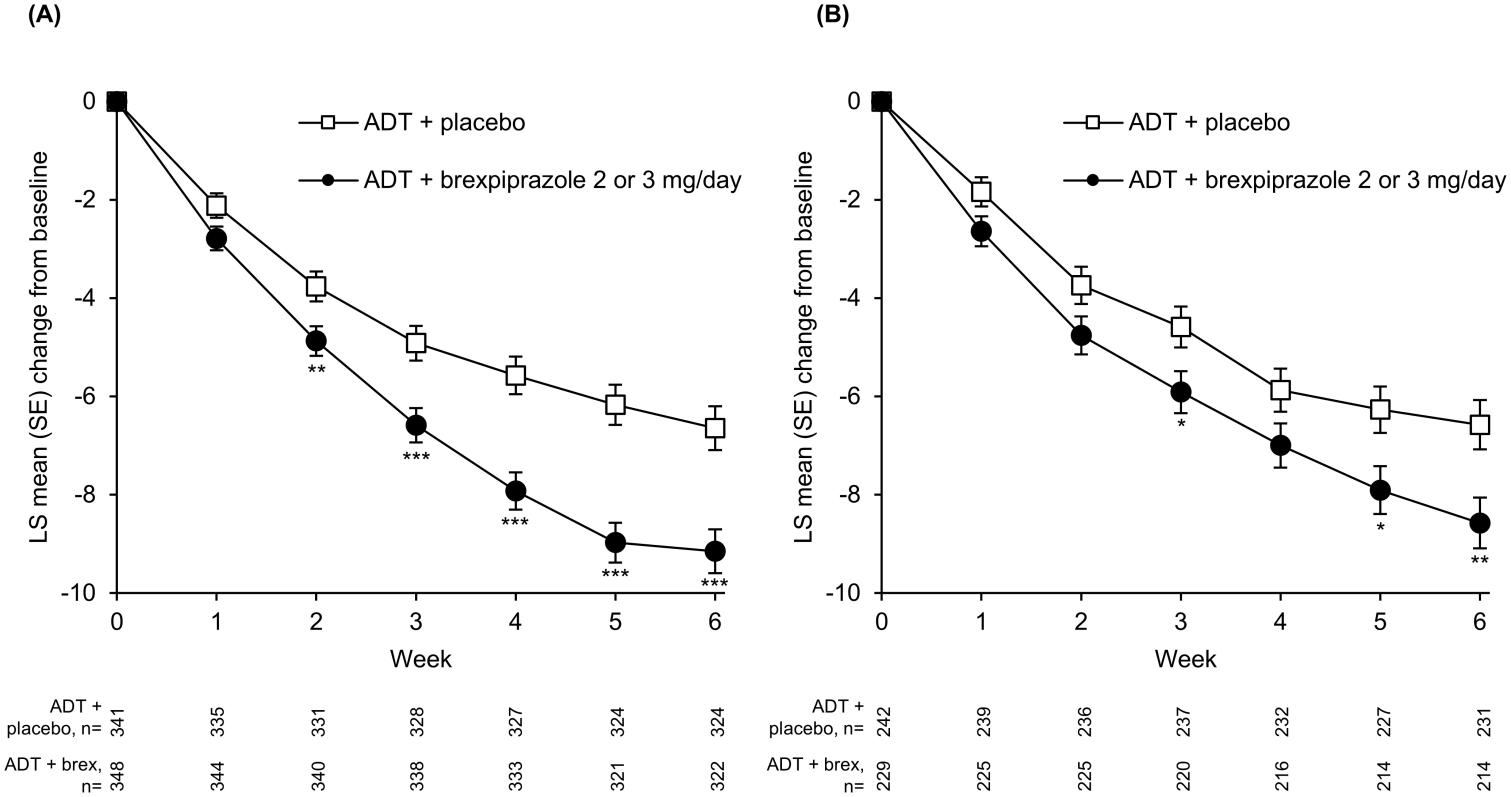
Mean change in MADRS Total score in **(A)** high SDF (≥4) and **(B)** low SDF (<4) subgroups. *p<0.05, **p<0.01, ***p<0.001 versus ADT + placebo; MMRM. Mean MADRS baseline score: high SDF subgroup: ADT + placebo, 27.8; ADT + brexpiprazole, 27.9; low SDF subgroup: ADT + placebo, 24.9; ADT + brexpiprazole, 25.1. ADT, antidepressant treatment; LS, least squares; MADRS, Montgomery–Åsberg Depression Rating Scale; MMRM, mixed model for repeated measures; SDF, Sleep Disturbance Factor; SE, standard error.

On depression outcomes, the LS mean change from baseline to Week 6 in MADRS Total score was greater with ADT + brexpiprazole than ADT + placebo (p<0.0001) in the high SDF subgroup. Greater improvement between treatment groups was observed from Week 2 (p<0.01) onwards (**Table 2**, **Figure 1A**). In the low SDF subgroup, greater improvement with ADT + brexpiprazole versus ADT + placebo was observed at Week 6 (p=0.0058) and at some earlier visits (**Table 2**, **Figure 1B**). LS mean changes from baseline to Week 6 in HAM-D_17_ Total score and CGI-S score were also greater with ADT + brexpiprazole versus ADT + placebo (p<0.05) in the high SDF subgroup and in the low SDF subgroup (**Table 2**). In the high SDF subgroup, MADRS response rates at Week 6 were 27.0% (94/348) with ADT + brexpiprazole and 20.8% (71/341) with ADT + placebo (p=0.032). In the low SDF subgroup, MADRS response rates were 29.3% (67/229) with ADT + brexpiprazole and 21.5% (52/242) with ADT + placebo (p=0.052).

**Table 2:** Summary of efficacy results in high SDF (≥4) and low SDF (<4) subgroups.

Endpoint	Sleep disturbance subgroup	Treatment group	N	Mean (SD) score at baseline	Mean (SD) score at Week 6	LS mean (SE) change from baseline to Week 6	LS mean difference (95% CI)	P-value	Cohen's *d* effect size
Depression outcomes									
MADRS Total score^a^	High SDF	ADT + brexpiprazole	348	27.9 (5.5)	18.7 (9.1)	-9.2 (0.4)	-2.50 (-3.74 to -1.27)	<0.0001	0.30
		ADT + placebo	341	27.8 (5.9)	21.0 (9.5)	-6.6 (0.4)			
	Low SDF	ADT + brexpiprazole	229	25.1 (5.1)	16.2 (8.1)	-8.6 (0.5)	-2.00 (-3.41 to -0.58)	0.0058	0.26
		ADT + placebo	242	24.9 (5.0)	18.4 (8.6)	-6.6 (0.5)			
HAM-D_17_ Total score^b^	High SDF	ADT + brexpiprazole	340	22.6 (3.7)	15.7 (6.8)	-6.6 (0.4)	-1.89 (-2.81 to -0.96)	<0.0001	0.31
		ADT + placebo	333	22.4 (3.7)	17.2 (6.6)	-4.7 (0.4)			
	Low SDF	ADT + brexpiprazole	224	19.6 (3.1)	13.2 (5.8)	-6.4 (0.4)	-1.45 (-2.57 to -0.33)	0.011	0.24
		ADT + placebo	238	19.3 (3.1)	14.6 (5.9)	-5.0 (0.4)			
CGI-S score^a^	High SDF	ADT + brexpiprazole	348	4.3 (0.6)	3.3 (1.1)	-1.0 (0.1)	-0.22 (-0.37 to -0.06)	0.0060	0.21
		ADT + placebo	341	4.3 (0.6)	3.5 (1.0)	-0.8 (0.1)			
	Low SDF	ADT + brexpiprazole	229	4.1 (0.6)	3.0 (1.0)	-1.1 (0.1)	-0.27 (-0.45 to -0.09)	0.0040	0.27
		ADT + placebo	242	4.1 (0.6)	3.3 (1.1)	-0.8 (0.1)			
Functioning outcome									
SDS score^a^	High SDF	ADT + brexpiprazole	339	5.9 (2.3)	4.5 (2.6)	-1.4 (0.1)	-0.40 (-0.72 to -0.07)	0.016	0.19
		ADT + placebo	328	6.0 (2.1)	4.9 (2.5)	-1.0 (0.1)			
	Low SDF	ADT + brexpiprazole	216	5.7 (2.1)	4.3 (2.4)	-1.4 (0.1)	-0.40 (-0.78 to -0.01)	0.046	0.19
		ADT + placebo	236	5.6 (2.0)	4.6 (2.6)	-1.0 (0.1)			
Sleep outcomes									
SDF score^b^	High SDF	ADT + brexpiprazole	340	4.9 (0.8)	3.5 (1.8)	-1.4 (0.1)	-0.30 (-0.56 to -0.05)	0.021	0.18
		ADT + placebo	333	4.8 (0.8)	3.7 (1.7)	-1.1 (0.1)			
MADRS “reduced sleep” score^a^	High SDF	ADT + brexpiprazole	348	3.8 (0.8)	2.7 (1.4)	-1.0 (0.1)	-0.35 (-0.54 to -0.16)	0.0003	0.28
		ADT + placebo	341	3.7 (0.9)	3.0 (1.4)	-0.7 (0.1)			

a MMRM.

b ANCOVA, LOCF.

ADT, antidepressant treatment; ANCOVA, analysis of covariance; CGI-S, Clinical Global Impression – Severity; HAM-D_17_, 17-item Hamilton Depression Rating Scale; LOCF, last observation carried forward; LS, least squares; MADRS, Montgomery–Åsberg Depression Rating Scale; MMRM, mixed model for repeated measures; SD, standard deviation; SDF, Sleep Disturbance Factor; SDS, Sheehan Disability Scale; SE, standard error.

Regarding functioning, the LS mean change from baseline to Week 6 in SDS score was greater with ADT + brexpiprazole versus ADT + placebo (p<0.05) in the high SDF subgroup and in the low SDF subgroup (**Table 2**).

In the high SDF subgroup, on sleep outcomes, the LS mean change from baseline to Week 6 in SDF score and in MADRS “reduced sleep” item score was greater with ADT + brexpiprazole versus ADT + placebo (p<0.05 for both measures) (**Table 2**).

### 3.3 Safety

The overall incidence of TEAEs was higher with ADT + brexpiprazole than ADT + placebo in the high SDF subgroup (59.8% compared with 51.6%) and in the low SDF subgroup (62.4% compared with 40.9%) (**Table 3**).

**Table 3:** Summary of treatment-emergent adverse events (TEAEs) in high SDF (≥4) and low SDF (<4) subgroups.

Event	High SDF subgroup		Low SDF subgroup	
	ADT + placebo (n=341)	ADT + brexpiprazole (n=348)	ADT + placebo (n=242)	ADT + brexpiprazole (n=229)
Patients with TEAEs, n (%)	176 (51.6)	208 (59.8)	99 (40.9)	143 (62.4)
Discontinuation due to AEs, n (%)	2 (0.6)	11 (3.2)	1 (0.4)	6 (2.6)
TEAEs occurring in ≥5% of patients in any subgroup, n (%)				
Akathisia	10 (2.9)	36 (10.3)	7 (2.9)	20 (8.7)
Weight increase	4 (1.2)	25 (7.2)	5 (2.1)	10 (4.4)
Headache	22 (6.5)	18 (5.2)	16 (6.6)	9 (3.9)
Restlessness	2 (0.6)	18 (5.2)	2 (0.8)	14 (6.1)
Somnolence	5 (1.5)	15 (4.3)	3 (1.2)	14 (6.1)
Upper respiratory tract infection	18 (5.3)	12 (3.4)	6 (2.5)	9 (3.9)
Other sedating TEAEs of interest, n (%)				
Fatigue	4 (1.2)	8 (2.3)	4 (1.7)	11 (4.8)
Sedation	1 (0.3)	2 (0.6)	1 (0.4)	1 (0.4)
Lethargy	1 (0.3)	1 (0.3)	0 (0)	1 (0.4)
Other activating TEAEs of interest, n (%)				
Insomnia	9 (2.6)	10 (2.9)	3 (1.2)	7 (3.1)
Initial insomnia	1 (0.3)	2 (0.6)	4 (1.7)	1 (0.4)
Middle insomnia	1 (0.3)	4 (1.1)	1 (0.4)	3 (1.3)
Terminal insomnia	0 (0)	1 (0.3)	0 (0)	3 (1.3)

ADT, antidepressant treatment; AE, adverse event; SDF, Sleep Disturbance Factor; TEAE, treatment-emergent adverse event.

In the high SDF subgroup, the TEAEs with an incidence ≥5% in the ADT + brexpiprazole group and greater than ADT + placebo were akathisia, weight increase, and restlessness (**Table 3**). In the low SDF subgroup, the TEAEs with an incidence ≥5% in the ADT + brexpiprazole group and greater than ADT + placebo were akathisia, restlessness, and somnolence (**Table 3**).

## 4 Discussion

In this pooled analysis of three randomized trials in MDD, adjunctive brexpiprazole was associated with greater improvements in depression symptoms (MADRS Total score, HAM-D_17_ Total score, CGI-S score) and functioning (SDS score) compared with adjunctive placebo, in patients with high and low baseline sleep disturbance. Additionally, in patients with high baseline sleep disturbance, adjunctive brexpiprazole was associated with greater improvement in sleep disturbance (SDF score, MADRS “reduced sleep” item score) compared with adjunctive placebo. These results support previous findings from an 8-week exploratory, flexible-dose, open-label study, in which sleep disturbances and depressive symptoms improved with adjunctive brexpiprazole in patients with MDD who had sleep disturbances (22, 23).

Effective management of sleep disturbances in MDD requires a balance between improving nighttime sleep quality and minimizing excessive daytime sedation (38). Many commonly used antidepressants, such as selective serotonin reuptake inhibitors, have been associated with worsening sleep disturbances, particularly insomnia (1). Additionally, while some atypical antipsychotics, such as quetiapine, may improve symptoms of sleep disturbances (measured by the SDF score) in patients with MDD (39, 40), their benefits may be a result of sedative effects (41). In the present analysis, the most common sedating TEAE with adjunctive brexpiprazole was somnolence in the low SDF subgroup (6.1%); all other sedating TEAEs occurred at an incidence of <5%. Prior analyses suggest that brexpiprazole is not a sedating (or activating) drug (42). Overall, augmentation strategies should be individualized depending on the requirements and preferences of each patient (15).

In the present analysis, there were no notable differences in TEAEs between the high SDF and low SDF subgroups. Regardless of SDF status, the most common TEAE with adjunctive brexpiprazole was akathisia (8.7–10.3%), as noted for the total sample in prior analyses (24). Weight increase was reported by 4.4–7.2% of patients on adjunctive brexpiprazole; prior analyses indicate that adjunctive brexpiprazole is associated with moderate weight gain (1.5 kg) over 6 weeks (43). Although akathisia and weight gain can potentially impact treatment adherence and tolerability (44, 45), the proportion of patients who discontinued adjunctive brexpiprazole due to adverse events was low (2.6–3.2%), indicating that the majority of patients tolerated treatment. Overall, no new safety signals were observed with adjunctive brexpiprazole in the present analysis (24).

The efficacy of adjunctive brexpiprazole in patients with sleep disturbances may be attributed to its receptor binding profile. Brexpiprazole has antagonist properties at 5-HT_2A_ receptors (46), which may promote slow-wave sleep, and be linked to cognitive performance and improved daytime functioning (47, 48). Additionally, brexpiprazole’s α_1_-adrenoceptor antagonism may enhance sleep quality (46, 49, 50) by reducing excessive noradrenergic activity, which has been associated with hyperarousal, disruptions in sleep and wakefulness, insomnia, and heightened states of alertness (51, 52).

Strengths of this analysis include the large dataset derived from three Phase 3, placebo-controlled, randomized trials. Limitations include its *post hoc* nature, meaning that results should be considered hypothesis generating, and the lack of adjustment for multiple comparisons, which may increase the risk of Type I error. The SDF, used as a proxy for sleep disturbances, includes insomnia-related items only (34), and cannot assess other aspects of sleep disturbance such as sleep architecture, hypersomnia, and circadian rhythm alterations. Additionally, clinical trial inclusion and exclusion criteria may limit generalizability of the results to broader patient populations. Further research is needed to validate these results in broader patient populations and to assess long-term effects.

In conclusion, in patients with MDD and an inadequate response to ADTs, adjunctive brexpiprazole was associated with improvements in depression and functioning regardless of baseline sleep disturbance, and improvement in sleep disturbances in patients with high baseline sleep disturbance. Improvement in sleep disturbance was generally not accompanied by TEAEs of daytime sedation, and no new safety signals were observed within each subgroup. Further prospective and long-term studies are needed to confirm these exploratory findings and to assess their generalizability to real-world settings. Nonetheless, given the challenges in managing sleep disturbances in MDD (1), these findings suggest that adjunctive brexpiprazole may be a valuable treatment option for patients with MDD and sleep disturbances.

## Data Availability

To submit inquiries related to Otsuka clinical research, or to request access to individual participant data (IPD) associated with any Otsuka clinical trial, please visit https://clinical-trials.otsuka.com/. For all approved IPD access requests, Otsuka will share anonymized IPD on a remotely accessible data sharing platform.
